# Anthraquinone G503 Induces Apoptosis in Gastric Cancer Cells through the Mitochondrial Pathway

**DOI:** 10.1371/journal.pone.0108286

**Published:** 2014-09-30

**Authors:** Lijun Huang, Ting Zhang, Shuai Li, Junting Duan, Fang Ye, Hanxiang Li, Zhigang She, Guoquan Gao, Xia Yang

**Affiliations:** 1 Department of Biochemistry, Zhongshan School of Medicine, Sun Yat-sen University, Guangzhou, Guangdong Province, China; 2 Key Laboratory of Functional Molecules from Marine Microorganisms (Sun Yat-sen University), Department of Education of Guangdong Province, Guangzhou, Guangdong Province, China; 3 China Key Laboratory of Tropical Disease Control (Sun Yat-sen University), Ministry of Education, Guangzhou, Guangdong Province, China; University of Navarra, Spain

## Abstract

G503 is an anthraquinone compound isolated from the secondary metabolites of a mangrove endophytic fungus from the South China Sea. The present study elucidates the anti-tumor activity and the underlying mechanism of G503. Cell viability assay performed in nine cancer cell lines and two normal cell lines demonstrated that the gastric cancer cell line SGC7901 is the most G503-sensitive cancer cells. G503 induced SGC7901 cell death via apoptosis. G503 exposure activated caspases-3, -8 and -9. Pretreatment with the pan-caspase inhibitor Z-VAD-FMK and caspase-9 inhibitor Z-LEHD-FMK, but not caspase-8 inbibitor Z-IETD-FMK, attenuated the effect of G503. These results suggested that the intrinsic mitochondrial apoptosis pathway, rather than the extrinsic pathway, was involved in G503-induced apoptosis. Furthermore, G503 increased the ratio of Bax to Bcl-2 in the mitochondria and decreased the ratio in the cytosol. G503 treatment resulted in mitochondrial depolarization, cytochrome c release and the subsequent cleavage of caspase -9 and -3. Moreover, it is reported that the endoplasmic reticulum apoptosis pathway may also be activated by G503 by inducing capase-4 cleavage. In consideration of the lower 50% inhibitory concentration for gastric cancer cells, G503 may serve as a promising candidate for gastric cancer chemotherapy.

## Introduction

Surgery, radiotherapy and chemotherapy are the primary clinical tumor treatments. However, surgery and radiotherapy are ineffective in metastatic cancer; if chemotherapy is used properly, metastasis may be inhibited [1]. With regard to anti-cancer drug development, anthracyclines are the most effective anti-cancer drugs [2]. Natural products from oceans are important sources of novel anti-cancer drugs [3]. Marine microorganism metabolites with unique structures and pharmacological activities are typically used as leading antitumor compounds [4]. Anthraquinone compounds, such as daunorubicin, doxorubicin, epirubicin and mitoxantrone, are the most effective clinical anti-cancer drugs [2]. The marine anthraquinone SZ-685C suppresses the proliferation of human breast cancer [5]–[6] and human nasopharyngeal carcinoma (NPC) cells [7]. Huang et al. demonstrated that anthraquinones from rhubarb, such as emodin, aloe-emodin and rhein, inhibit the growth and proliferation of various cancer cells, such as lung adenocarcinoma, myelogenous leukemia, neuroblastoma, hepatocellular carcinoma, bladder cancer and others [8].

The mechanism of the antitumor activity of anthraquinones is primarily involved in the following aspects [9]–[10]: DNA interactions through binding, intercalating and interfering separation of the DNA double strand; direct membrane effects; DNA damage in response to topoisomerase II inhibition or the generation of free radicals, such as reactive oxygen species (ROS), and the induction of apoptosis via topoisomerase II inhibition, functional p53 or ROS generation. In addition, anthraquinones also trigger apoptosis through the JNK(c-Jun N-terminal kinase) [11], Akt/PKB (Protein Kinase B) [5], [12] and mitochondrial pathways [13]–[14].

G503 is an anthraquinone compound isolated from the secondary metabolites of the mangrove endophytic fungus No. 1403 from the South China Sea [15].However, whether G503 possesses anticancer potential as an anthraquinone compound has not been investigated. Therefore, the present study was designed to elucidate the anti-tumor activity of G503 and the underlying mechanism involved.

## Materials and Methods

### Chemicals and Reagents

3-(4,5-dimethylthiazol-2-yl)-2,5-diphenylterazolium bromide (MTT) and ROS scavenger N-acetyl-cysteine (NAC) were purchased from Sigma-Aldrich (St. Louis, MO, USA). Hochest 33342, carboxy-H2DCFDA, MitoProbe DiOC2 (3) Assay Kit and MitoProbe Transition Pore Assay Kit (M34153) were obtained from Invitrogen (USA). Annexin V-FITC (fluorescein isothiocyanate)/PI (propidium iodide) Apoptosis Detection Kit was purchased from Keygen (Nanjing, Jiangsu, CHINA). RPMI1640 medium and fetal bovine serum (FBS) were from Hyclon (Logan, UT, USA*).* Caspase-8 inhibitor Z-IETD-FMK, caspase-9 inhibitor Z-LEHD-FMK and caspase-family inhibitor Z-VAD-FMK were from Calbiochem (Gibbstown, NJ, USA) and Beyotime (CHINA). Antibodies against caspase-3, caspase-4, caspase-8, caspase-9 and COXIV antibody were from Cell Signaling Technology (Beverly, MA, USA). Antibodies against cytochrome *c*, Bax, Bcl-2, p38, p-p38 and anti-goat LgG-HRP were from Santa Cruz Biotechnology (Santa Cruz, CA, USA). Mouse anti-β-actin and anti-GAPDH primary antibodywere from Sigma-Aldrich (St. Louis, MO, USA). Anti-rabbit LgG-peroxidase and anti-mouse IgG-peroxidase were from Vector (Burlingame, CA, USA).Mitochondria isolation kit was purchased from Pierce (Pierce, IL, USA), protein assay kit were purchased from Bio-Rad (Hercules, CA, USA), and the ECL detection kit were from Applygen Technologies Inc (Beijing, CHINA).

### Fermentation, extraction and isolation of G503 from *Nigrospora*sp. No. 1403

NO.1403 was isolated from decayed wood of *Kandeliacandel* (L.) Druce and reidentified as *Nigrospora* sp. (Genebank accession number: HQ891110), collected from Mai Po, Hong Kong, and a salt lake in the Bahamas. G503 was isolated and purified from the secondary metabolites of NO. 1403 [15]. Starter cultures were maintained on cornmeal seawater agar. Plugs of agar supporting mycelial growth were cut and transferred aseptically to a 250 mL Erlenmeyer flask containing 100 mL of liquid medium (glucose 10 g/L, peptone 2 g/L, yeast extract 1 g/L, NaCl_2_ g/L, pH D 7.0). The flask was incubated at 28°C. After shaking on a rotary shaker for 3–5 days, the mycelium was aseptically transferred to a 500 ml Erlenmeyer flask containing the culture liquid (200 mL). The flask was then incubated at 28°C for 25 days.

The cultures (150 L) were filtered through cheesecloth. The filtrate was concentrated to 5 L below 50°C and extracted three times by shaking with an equal volume of ethyl acetate. The combined organic extracts were subjected to a silica gel column, and eluted with a gradient of petroleum ether to ethyl acetate to yield the compound.

The compound was dissolved in dimethyl sulfoxide (DMSO) at a stock concentration of 50 mmol/L and diluted to specific concentrations prior to use.

### Cell culture

HUVECs were isolated from the human umbilical vein cords of normal parturitions and cultured following a protocol described previously [16]–[17]. Chang liver cells and tumor cell lines HONE-1, CNE-2, 5–8F, HepG2, B7402, Rb, PC3, A549 and SGC7901, AGS were maintained by our laboratory and cultured in RPMI1640 medium supplemented with 10% FBS, 100 U/mL streptomycin and 100 U/mL penicillin (Gibco) at 37°C in a humidified atmosphere of 5% CO_2_. This study conforms to the principles outlined in the Declaration of Helsinki, approved by the Medical Ethics Committee of Sun Yat-Sen University, and written informed consent was obtained from the donor.

### Cell viability assay

Cells were seeded at a density of 2×10^4^ cells/mL in 24-well plates (HUVECs were seeded in gelatin-coated 24-well plates). When the cells achieved a density of 60%–70%, they were treated with various concentrations of G503 for 48 h in RPMI1640 medium (1 mL/well) without FBS; the negative control groups were treated with PBS. Subsequently, 100 µL MTT (5mg/mL) dissolved in PBS was added to each well and incubated for an additional 4 h to allow for the formation of formazancrystals. The crystals were solubilized in 1 mL DMSO in each well. The optical density (OD) values of the purple solution that represented cell viability were measured at 570nm [18]–[19]. After three independent experiments, cell survival was calculated using the following formula: survival (%) = (mean experimental OD value/mean control OD value) ×100%. The values were expressed as the 50% inhibitory concentration (IC_50_), which was calculated by the regression method in the linear range.

### Hoechst 33342 staining assay

SGC7901 cells were plated in 6-well plates at a density of 10^5^ cells per well and treated with G503 concentrations ranging from 0 µmol/L to 40 µmol/L for 24 h. The cells were labeled with 10 µg/mL Hoechst 33342 [20] for 10 min at 37°C and observed using fluorescence microscopy (Olympus X71-A12FL/PH).

### Annexin V-FITC/PI staining assay

SGC7901 cells were collected at a density of 5×10^5^ to 5×10^6^/mL after G503 treatment at concentrations ranging from 0 µmol/L to 40 µmol/L for 24 h in 6-well plates; cells treated with 25 µmol/L colchicine were used as positive control. The cells were then washed and stained according to the manufacturer’s instructions (Annexin V-FITC/PI Apoptosis Detection Kit). Briefly, the cells were resuspended in 500 µL 1×binding buffer. Next, 5 µL AnnexinV-FITC and 5 µL 20 µg/mL PI was added to the sample and incubated at room temperature for 15 min in the dark. The stained samples were then assessed by flow cytometry (Becton Dickinson, NJ, USA) to identify apoptotic cells.

### Determination of mitochondrial membrane potential

SGC7901 cells were plated in 6-well plates. Upon achieving a density of 60%, the cells were treated with 20 µmol/L G503 for 18 h and then collected to assess the mitochondrial membrane potential according to the manufacturer’s recommendations(MitoProbe DiOC2 (3) Assay Kit). Briefly, 1×10^6^/mL cells were resuspended in 37°C PBS. The positive control groups were pre-treated with 1 µL of 50mmol/L CCCP at 37°C for 5 min. All of the groups were then treated with 5 µL of 10 µmol/L DiOC_2_ (3) for 30 min at 37°C and assessed by flow cytometry. The mitochondrial membrane potential was calculated based on the following equation: mitochondrial membrane potential = (red fluorescence intensity)/(green fluorescence intensity).

### Detection the opening of mitochondria Permeability Transition Pore (mPTP)

SGC7901 cells were plated in 6-well plates. Upon achieving a density of 60%, the cells were treated with 20µmol/L G503 for 18h and collected to assess mPTP opening by flow cytometry (Becton Dickinson, NJ, USA) according to the manufacturer’s protocol (MitoProbe Transition Pore Assay Kit). Briefly, each sample was divided into 3 aliquots and treated as follows: aliquot 1 was treated with 5 µL 2 µmol/L Calcein AM in the dark; aliquot 2 was treated with 5 µL Calcein AM and 5 µL 80 mmol/L CoCl_2_ in the dark; and aliquot 3 was treated with 5 µL Calcein AM, 5 µL CoCl_2_ and 5 µL of 100 µmol/L ionomycin. The aliquots were then incubated at 37°C for 15 min in the dark. After being washed with HBSS/Ca^2+^ and re-suspended in 400 µL HBSS/Ca^2+^, the cells were assessed by flow cytometry within 1 h. The mPTP opening condition is calculated as follows: (fluorescence of aliquot 2– the fluorescence of aliquot 3)/(the fluorescence of aliquot 1– the fluorescence of aliquot 3). Calcein AM penetrates the cytoplasm and organelles including the mitochondria. CoCl_2_ quenches Calcein AM fluorescence in the cytoplasm but not in the mitochondria. Calcein AM translocates to the cytoplasm from the mitochondria when the mPTP is opened, and the fluorescence is attenuated by CoCl_2_.

### Isolation of cell mitochondria and cytoplasm

SGC7901 cells were plated into 100-mm plates; each group was plated into two plates. After achieving 60%–70% confluence, the cells were treated with or without 20 µmol/L G503 for 18 h. After treatment, the mitochondrial and cytoplasmic fractions were obtained according to the manufacturer’s instructions (Mitochondria isolation kit).

### Western blotting analysis

As described previously, the cells were lysed in RIPA buffer [21]. The protein concentrations of the mitochondria, cytoplasm and whole-cell were detected by the BCA method using the Bio-Rad protein assay kit. In total, 60 µg proteins from each group were separated by 12% or 15% sodium dodecylsulfate-polyacrylamide gel electrophoresis (SDS-PAGE). The proteins from SDS-PAGE were transferred to a PVDF membrane. After the nonspecific binding sites of the membranes were blocked for 1–2 h at room temperature with TBST buffer containing 10% non-fat milk, the membranes were incubated overnight at 4°C with primary antibody according to the manufacturer’s instructions. The secondary antibodies with or without fluorescence conjugated to the relevant primary antibodies were incubated for 2 h at room temperature. The membranes with fluorescent secondary antibodies were assessed using the Odyssey system (Li-COR, Nebraska,USA) to scan the fluorescent bands [22], and the membranes with normal secondary antibodies were visualized using the ECL detection kit for immunoblots. The membranes were stripped and re-probed with β-actin or COXIV antibodies. β-actin was used as the internal standard for the total cell lysate and cytoplasmic extractions, whereas COXIV was used as the control for the mitochondrial extractions. Densitometric analysis of bands was performed using Quantity One (Bio-Rad, Hercules, CA, USA) [23].

### Statistical analysis

All data were presented as means ± SD at least triplicate determinations. SPSS 13.0 software was used for Student’s t-test and one-way analysis of variance (ANOVA) in all statistical analyses. A *P* value 0.05 was considered statistically significant in all cases.

## Results

### G503-mediated inhibition on proliferation is the most potent in SGC7901 cells among the 11 cell lines examined

We used the MTT assay to determine whether the anthraquinone compound G503 is cytotoxic in the following cell lines: two normal cell lines (Human umbilical vein endothelial cells (HUVECs) and Chang liver cells and nine tumor cell lines (HONE-1, CNE-2, 5–8F, HepG2, B7402, Rb, PC3, A549 and SGC7901). All cells were incubated with 0, 2.5, 5, 10, 20 or 40 µmol/L G503 for 48 h. Cell viabilities were measured using the MTT assay as noted, and the IC_50_ value for SGC7901 cells was the lowest, whereas the IC_50_ values for the normal cell lines, HUVEC and Chang liver cells, were significantly greater than SGC7901 (Table 1).

**Table 1:** IC_50_ values of G503 on cancer cell lines and normal cell lines.

Cell line	IC_50_ (µmol/L)
HONE-1	35.7
CNE-2	35.6
5–8F	31.7
A549	20.0
HepG2	13.3
B7402	19.8
Rb	44.0
PC3	21.1
SGC7901	10.24
HUVEC	22.4
Chang liver cells	17.5

### G503 induces apoptosis in SGC7901 and AGS gastric cancer cells

**Figure 1:**
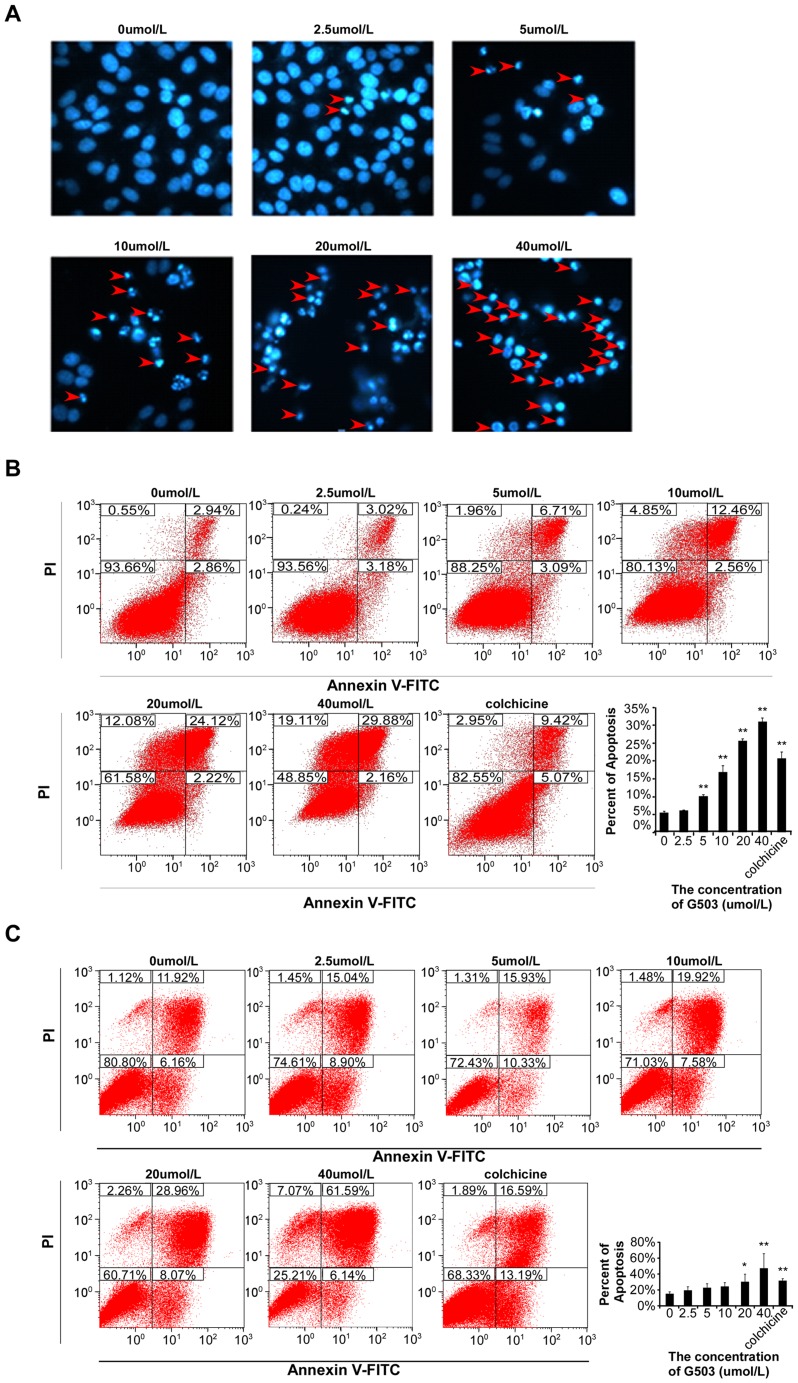
The effects of various G503 concentrations on gastric cancer cell apoptosis. (A) SGC7901 cells were treated with various G503 concentrations (0–40 µmol/L) for 24 h and stained with Hoechst 33342. Red arrows indicate the apoptotic cells. (B),(C) SGC7901 cells (B) and AGS cells (C) were analyzed by flow cytometry after treatment with various G503 concentrations- for 24 h. For the quantitative analysis of G503-induced SGC7901 and AGS cell apoptosis, all data are presented as the mean ± SD of three independent experiments. * and ** represent *P*<0.05 and *P*<0.01, respectively.

Given that the SGC7901 cell line was the most sensitive to G503 of the eleven cell lines examined (Table 1), we further investigated this cell line separately. SGC7901 cells were stained with Hoechst 33342 to determine whether the inhibitory effect was related to apoptosis. The results indicate an increase in the number of cells displaying nuclear shrinkage and chromatin condensation with increasing G503 concentrations (Figure 1A). In addition, to investigate whether G503 induces apoptosis in other gastric cancer cell lines, AGS gastric cancer cells were also examined. Annexin V/PI staining was employed to analyze the effects of G503 in SGC7901 and AGS gastric cancer cells by flow cytometry. For these experiments, 25 µmol/L colchicine was used as positive control. After treatment with 0 µmol/L, 2.5 µmol/L, 5 µmol/L, 10 µmol/L, 20 µmol/L and 40 µmol/L G503 and 25 µmol/L colchicines for 24 h, the percentage of apoptotic SGC7901 cells were 5.625% ±0.50%, 6.19% ±0.13%, 10.25% ±0.47%, 16.99% ±1.73%, 25.72% ±0.55%, 31.03±1.03% and 20.69% ±1.91%, respectively (Figure 1B); the percentage of apoptotic AGS cells were 15.37% ±2.36%, 19.30% ±4.45%, 22.72% ±4.89%, 24.93% ±4.76%, 30.44% ±9.42%, 47.51% ±18.29% and 31.49% ±2.45%, respectively (Figure 1C). The detection of apoptosis in AGS cells and the above results indicated that G503 induces gastric cancer cell apoptosis in a dose-dependent manner.

### G503 induces apoptosis in a caspase-dependent manner

**Figure 2:**
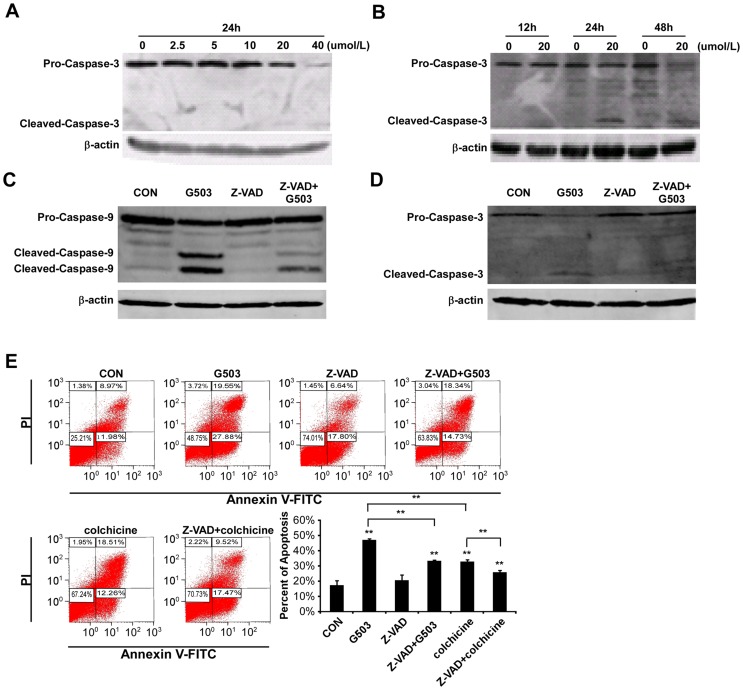
The effects of G503 and Z-VAD-FMK on caspase activation and apoptosis in SGC7901 cells. (A),(B) Cells were treated with various G503 concentrations for 24 h (A) and treated with 20 µmol/L G503 for various time (B). All celluar proteins were extracted and caspase-3 protein levels were analyzed by Western blotting. (C)–(E) Cells were pre-incubated with Z-VAD-FMK for 30 min before treated with 20µmol/L G503 for 24 h. All celluar proteins were extracted and caspase-9 (C), −3 (D) protein levels were analyzed by Western blotting. The cellular apoptotic rates were determined by flow cytometry (E). All values are displayed as the mean ± SD of at least three independent experiments (*P<0.05, **P<0.01 vs. control).

To further investigate the mechanism involved in G503-induced apoptosis in gastric cancer cells, we studied SGC7901 cells. Caspase-3 is an effector caspase that can enter nucleus and directly interact with its substrate, thereby promoting cell apoptosis [24]. Caspse-9 is the initiator caspase that activates caspse-3 [25]. The cells were treated with various G503 concentrations for indicated times and collected to assess caspase-3 and -9 by Western blotting. The expression of the caspase-3 precursor decreased in a dose- and time-dependent manner, whereas caspase-3 cleavage fragments increased in a dose- and time-dependent fashion (Figure 2A, B). The expression of the caspase-9 precursor also decreased and the cleavage fragments increased after the cells were treated with 20 µmol/L G503 for 24 h (Figure 2C). These effects are abolished by the pan-caspase inhibitor Z-VAD-FMK (Figure 2C, D). Consistent with the activation of caspase-9 and -3, the apoptotic cell rates induced induced by 20 µmol/L G503 in SGC7901 cells were markedly reduced after pretreatment with Z-VAD-FMK (Figure 2E). These data suggested that G503 induces apoptosis in SGC7901 cells in a caspase-dependent manner.

### G503-induced apoptosis is not dependent on caspase-8

**Figure 3:**
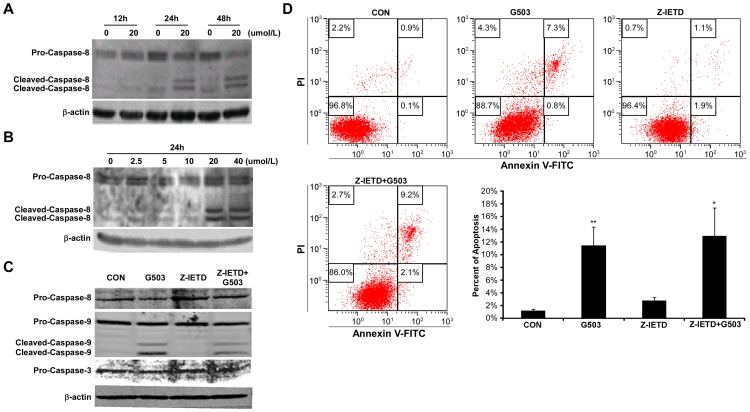
The effects of G503 and Z-IETD-FMK on caspase activation and apoptosis in SGC7901 cells. (A), (B) Cells were treated with 20µmol/L G503 for various times (A) and treated with various G503 concentrations (0–40 µmol/L) for 24 h (B). The cellular total proteins were extracted to detect the levels of caspase-8 by Western blotting. (C), (D) Cells were pre-incubated with 20 µmol/L Z-IETD-FMK for 30 min and subsequently treated with 20 µmol/L G503 for 24 h. The cellular proteins were extracted to detect the levels of caspase −8, −9 and −3 by Western blotting (C). The cellular apoptotic rates were determined by flow cytometry (D). All values are presented as the mean ± SD of at least three independent experiments (*P<0.05, **P<0.01 vs. control).

To investigate whether the death receptor-mediated apoptotic pathway is induced by G503, we studied caspase-8, the initiator caspase of the death receptor-mediated apoptotic pathway [26]. SGC7901 cells were treated with different doses of G503 for indicated times. The cells were collected to measure caspase-8 by Western blotting. The results indicated that caspase-8 precursor decreased and cleaved caspase-8 increased in a time- and dose-dependent manner (Figure 3A, B). The cells were pre-treated with 20 µmol/L caspase-8 inhibitor Z-IETD-FMK for 30 min and then treated with 20µmol/L G503 for 24 h. The caspase-8 proform increased in cells co-treated with the caspase-8 inhibitor and G503 compared with cells treated with G503 only. However, the caspase-9 proform, cleaved caspase-9 and the caspase-3 were maintained at similar levels in cells treated with the caspase-8 inhibitor and G503 or G503 alone (Figure 3C). Consistent with Figure 3C, G503-induced apoptotic cell rates in SGC7901 cells were not reduced by pretreatment with Z-IETD-FMK (Figure 3D). These data suggested that despite of activation by G503, caspase-8 is not involved in pro-apoptotic activity of G503.

### G503-induced apoptosis is dependent on caspase-9

**Figure 4:**
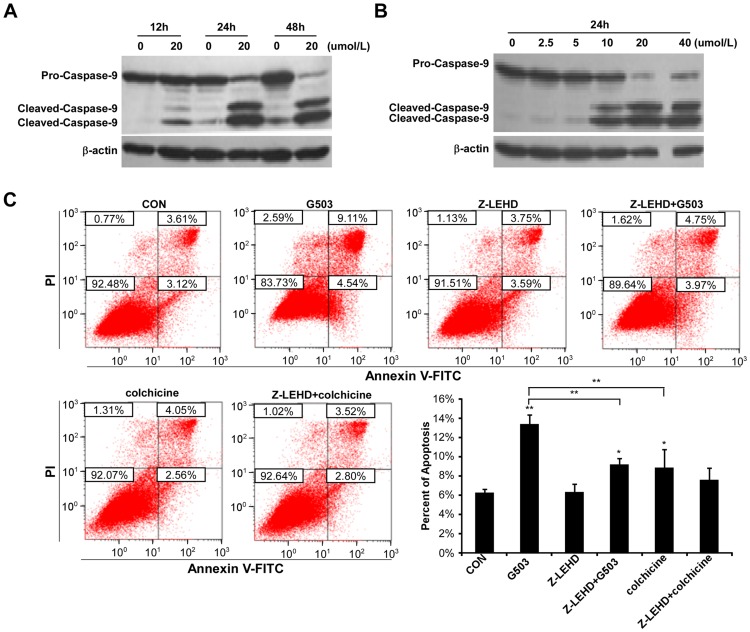
The relationship between caspase-9 and G503-induced apoptosis in SGC7901 cells. (A),(B) Cells were treated with 20µmol/L G503 for various times (A) and treated with various concentrations of G503 for 24 h (B). Cellular proteins were ectracted and caspase-9 protein levels were analyzed by Western blotting. (C) Cells were pre-incubated with Z-LEHD-FMK for 30 min followed by 20 µmol/L G503 for 24 h. The cellular apoptotic rate was detected by flow cytometry. All values are presented as the mean ± SD of at least three independent experiments. *and ** denote *P*<0.05 and *P*<0.01, respectively.

Caspase-9, the initiator caspase of the mitochondrial apoptotic pathway, can be activated by combining with cytochrome *c* (Cyto *c*) and apoptotic protease-activating factor-1 (Apaf-1) [25]–[26]. To further investigate whether the mitochondrial pathway is involved in G503-induced apoptosis, the activation of caspase-9 was examined after G503 treatment at various concentrations and times. In addition, the apoptotic cell rate was also measured upon co-treatment with G503 and caspase-9 inhibitor Z-LEHD-FMK. The results indicated that the caspase-9 proform decreased and the cleavage fragments increased in a time- and dose-dependent manner (Figure 4A, B). Next, the cells were pre-treated with 20 µmol/L Z-LEHD-FMK for 30 min and co-incubated with 20 µmol/L G503 for 24 h. To quantify the apoptotic cells, the cells were stained with AnnexinV/PI and analyzed by flow cytometry. The results indicated that the apoptotic cell rate decreased when the cells were co-treated with Z-LEHD-FMK and G503 (Figure 4C). Taken together, these data indicated that G503-induced SGC7901 cell apoptosis is dependent on caspase-9 activation.

### G503 induces apoptosis via the mitochondrial apoptotic pathway

**Figure 5:**
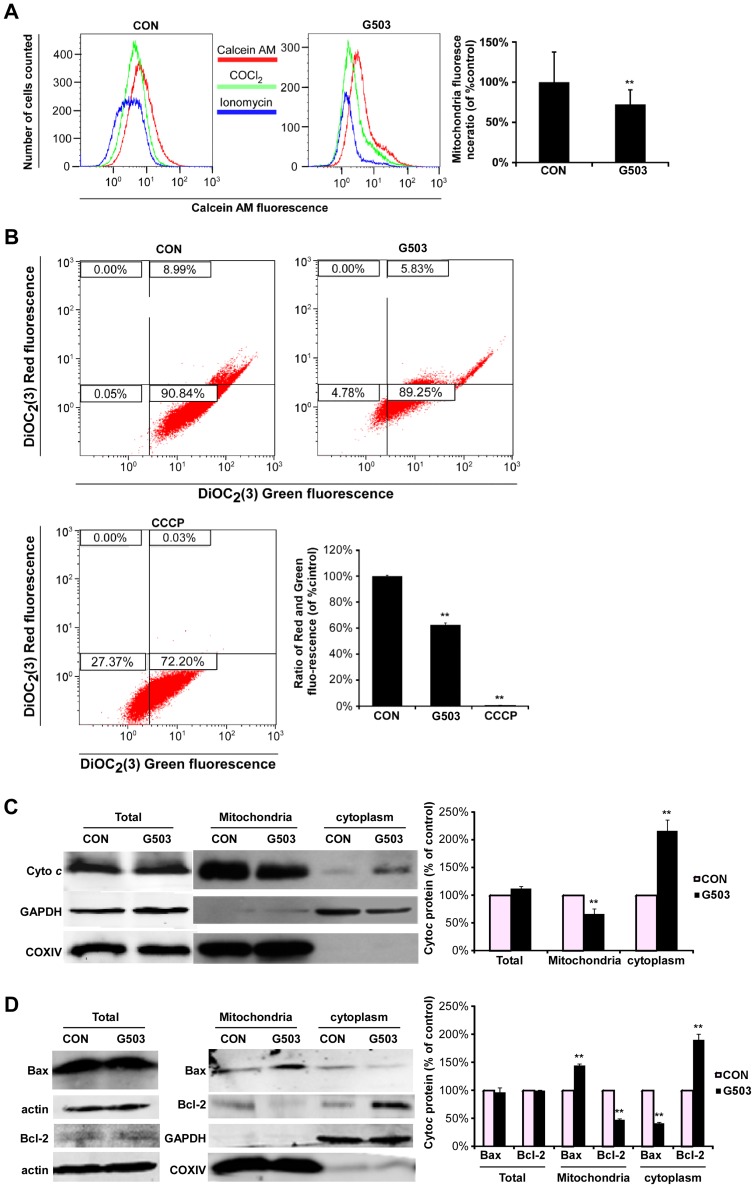
G503 promotes the activation of the mitochondrial apoptotic pathway in SGC7901 cells. (A) Cells were treated with 20 µmol/L G503 for 18 h, and mPTP opening was detected using the mitochondrial membrane permeability fluorescence dye Calcein AM and flow cytometry. (B) Cells were treated with 20 µmol/L G503 for 18 h, and the membrane potential was detected using flow cytometry. “CCCP” is the positive control reagent in the assay kit. (C), (D) Cells were treated with G503 for 18 h. Total protein extracts were collected for Western blotting to assess the levels of Cyto *c* (C) and Bax/Bcl-2 (D) in the total protein, mitochondrial and cytoplasmic fractions. All values are presented as the mean ± SD of at least three independent experiments (*P<0.05, **P<0.01 vs. control).

It is well known that the mitochondrial pathway is an important apoptotic pathway. To investigate whether G503 induces apoptosis via the mitochondrial pathway, we examined apoptosis-related proteins, the mitochondrial membrane potential (MMP), and the opening of mPTP to confirm the induction of the mitochondrial apoptotic pathway. Follow by G503 treatment, mitochondrial fluorescence decreased compared with control group (Figure 5A), thereby suggesting that the mPTP was opened by G503. Moreover, as shown in Figure 5B, considerable red/green fluorescence intensity was observed in the control group. However, after treatment with 20 µmol/L G503, the red/green fluorescence intensity was reduced. This result suggested that G503 decreases the mitochondrial membrane potential of SGC7901 cells. In addition, total Cyto *c* protein levels did not notably change between the control and drug group; however, Cyto *c* was relocalized to the cytoplasm from the mitochondria (Figure 5C). Bax and Bcl-2 are crucial factors in the regulation of mitochondrial channels and the release of various apoptosis-related proteins [27]. Therefore, We studied the distribution of Bax and Bcl-2 in different cellular compartments and did not observe a prominent change in the total protein levels of Bax and Bcl-2 between the control and drug group; however, G503 promoted the translocation of Bax from the cytoplasm to the mitochondria as well as the translocation of Bcl-2 from the mitochondria to the cytoplasm (Figure 5D). Taken together, G503 induces apoptosis in SGC7901 cells via the mitochondrial pathway.

### G503 induces apoptosis in SGC7901 cells in part through caspase-4 activation

**Figure 6:**
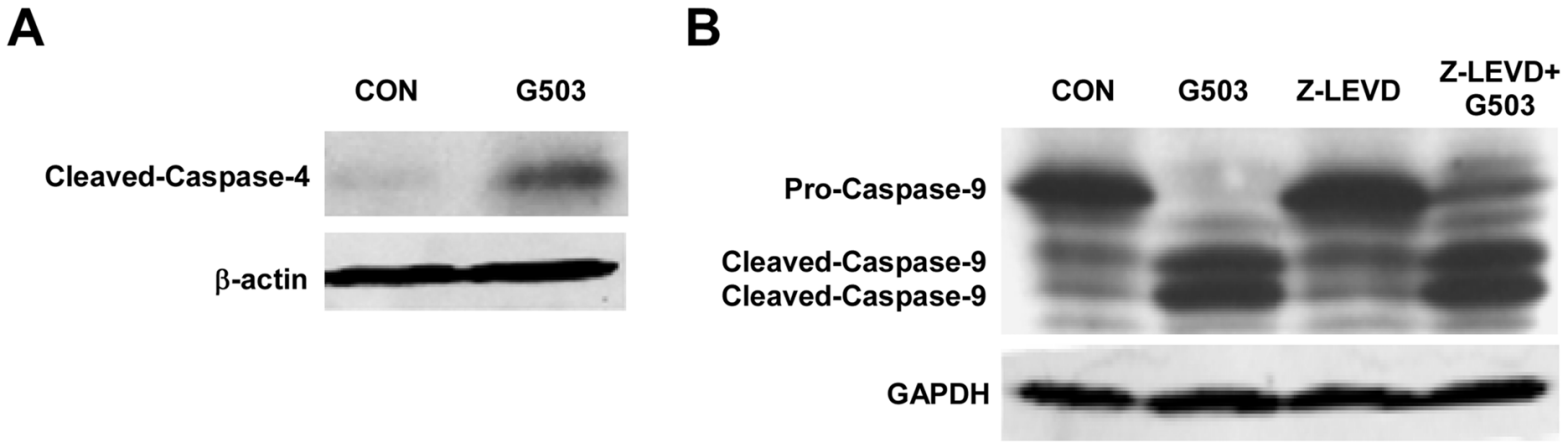
The relationship between caspase-4 and G503-induced apoptosis in SGC7901 cells. (A) Cells were treated with 20 µmol/L G503 for 24 h, and total protein extracts were collected for Western blotting. (B) SGC7901 cells were pre-incubated with 20 µmol/L Z-LEVD-FMK for 30 min followed by 20 µmol/L G503 for 24 h. The cellular protein extracts were used to detect caspase-9 levels by Western blotting.

Caspase-4 can directly activate caspase-9 but not through the mitochondrial pathway [28]–[29]. To investigate whether caspase-4 is activated by G503 and whether activated caspase-4 activates caspase-9, we examined the cleavage fragments of caspase-4 and -9. The results indicated that the caspase-4 cleavage fragment increased significantly in cells treated with G503 compared with the control group (Figure 6A). Treatment with the caspase-4 inhibitor Z-LEVD-FMK increased the caspase-9 proform, which was not detected in the G503 treatment group (Figure 6B). These results indicated that G503 induces SGC7901 gastric cancer cell apoptosis in part through the activation of caspase-4.

## Discussion

It is reported that *p*-quinone moiety of quinone-containing molecular plays an very important role in anti-cancer potential [30]. Perchellet et al. also found that p-quinone J4, o-quinone J1 and unsubstituted model p-quinone J7 reduced the most L1210 tumor cell viability among eight compounds synthesized and screened in their preliminary study [31]. To understand the relationship between the anti-cancer potential of altersolanol A and structure-activity, Teiten et al. evaluated the effect of altersolanol A and its related anthracene derivatives: tetraacetylaltersolanol A, tetrahydroaltersolanol B and ampelanol. They found that only altersolanol A and its acetylated derivative tetraacetylaltersolanol A present a cytotoxic effect on K562 cells, whereas derivatives with reduction of one of the carbonyl groups, such as tetrahydroaltersolanol B and ampelanol, have no effect [32]. The *p*-quinone structure of G503 (Figure S1) may also be contributed to cancer cell apoptosis, and in the next research we will analyze the G503 and its derivatives to identify the preliminary structure-activity relationship. In this study, we find that the gastric cancer cell line SGC7901 is the most G503-sensitive cancer cell line examined using a cell viability assay in nine cancer cell lines and two normal cell lines. G503 induces SGC7901 gastric cancer cell death via apoptosis in a dose-dependent manner. Another gastric cancer cells AGS is also examined, and is sensitive to G503-induced apoptosis as well. As a result,20 µmol/L G503 is the optimum concentration that induces severe apoptosis.

The human genome encodes at least 10 types of caspase homologs. The following caspase homologs participate in apoptosis: Caspase-2, -3, -8, -9 and -10 [33]. Although caspases play a role in apoptosis, there are various caspase independent pathways exist. For example, in the presence of caspase inhibitors, tumor necrosis factor (TNF) induces cell necrosis, which possesses the characteristics of apoptosis and necrosis [34]–[36]. In addition, apoptosis-inducing factor (AIF) is a DNA enzyme that directly degrades DNA. When the mitochondrial membrane potential is altered, AIF is released to the cytoplasm from the mitochondria and is activated by its interaction with cyclophilinA [37]–[38]. Endo G can also degrade DNA directly in a caspase-independent manner [39].

In our study, G503 exposure activates caspase-3, -8 and -9 in a dose- and time-dependent manner (Figure 2A, B; 3A, B; 4A, B). Is G503-induced apoptosis dependent on caspases? When cells were co-treated with Z-VAD-FMK and G503, Z-VAD-FMK inhibits caspase-9 and -3 activation (Figure 2C, D), and the number of apoptotic cells induced by G503 decreased (Figure 2E), thereby indicating that G503-induced SGC7901 cell apoptosis is caspase-dependent. To distinguish whether G503-induced apoptosis is dependent on caspase-8, -9 or both, SGC7901 cells were pretreated with Z-IETD-FMK and Z-LEHD-FMK and then co-treated with G503. The results indicate that Z-IETD-FMK do not inhibit caspase-9 and -3 activation (Figure 3C) nor decrease the number of apoptotic cells induced by G503 (Figure 3D); however, Z-LEHD-FMK decreased the number of apoptotic cells (Figure 4C). Thus, G503-induced SGC7901 cell apoptosis is not dependent on caspase-8 but is on caspase-9.

The mitochondria consist of an outer membrane, membrane space and matrix. Apoptosis-related proteins, such as caspase proenzymes, Cyto *c* and AIF exist in the membrane space. However, when pores formed in the outer mitochondrial membrane or the permeability is altered, these apoptosis-related proteins are released to the cytoplasm. Members of the Bcl-2 family control the permeability of the outer mitochondrial membrane, and can be divided into two members that promote apoptosis (such as Bax and Bak) or inhibit apoptosis (such as Bcl-2 and Bcl-xL). The pro-apoptotic members promote the release of apoptosis-related proteins, such as Cyto *c*, from the mitochondria, whereas the anti-apoptotic members have an opposite effect [40]. Bax primarily exists in the cytoplasm, whereas Bcl-2 combines with hydrophobic groups at the C-terminal of the cell’s bio-membrane [41]. Bax structure is altered during external stress so that it combines with the outer mitochondrial membrane tospontaneously form a gallery by homologous oligomerization. This action induces the release of apoptosis-related proteins [42] or the formation of the mitochondria Permeability Transition Pore (mPTP), which consists of voltage-dependent anion channel (VDAC) on the outer mitochondrial membrane and adenine nucleotide translocator (ANT) on the inner membrane [43]. Bax only interacts with VDAC to create an open pore when Bax concentrations are low. In contrast, Bax simultaneously interacts with VDAC and ANT when Bax concentrations are high to open the pores in the inner and outer membrane. This action reduces the inner membrane potential, increases the osmotic pressure of the mitochondrial matrix, promotes inner membrane swelling and increases the osmotic pressure of the outer membrane, thereby promoting the release of apoptosis-related proteins [26], [44]–[46]. An important step in the mitochondrial apoptotic pathway is the release of Cyto *c* [47]–[49]. Cyto *c* combines with Apaf-1 and the caspase-9 proform to create the apoptosome. Apaf-1 exposes its oligomerization domain, and the N-terminal Caspase activation and recruitment domains (CARD) combines with the caspase-9 proform CARD. Thus, caspase-9 is activated, and the activated caspase-9 cleaves downstream caspases, such as caspse-3 and -7. The effector caspases eventually cleave their corresponding substrates to induce cell apoptosis [47], [49]–[50].

In this study, G503 increases the proportion of Bax/Bcl-2 on the mitochondrial membrane and decreases the Bax/Bcl-2 ratio in the cytoplasm (Figure 5D). G503 also promotes mPTP opening and the reduction of the mitochondrial membrane potential (Figure 5A, B). Then, Cyto *c* was transferred from the mitochondria to the cytoplasm (Figure 5C). In addition, G503 promotes the transfer of Bax to the mitochondria from the cytoplasm and Bcl-2 to the cytoplasm from the mitochondria. Increased Bax and reduced Bcl-2 in the mitochondria increase the permeation of the mitochondrial outer membrane and promote the release of Cyto *c*. The regulation of Bax/Bcl-2 by G503 may be observed because G503 is a small molecular compound. It is possible that G503 directly enters cells and combines with Bax and Bcl-2 to alter their structures, thereby causing Bax binding to the outer mitochondrial membrane and Bcl-2 release from the mitochondria.

Several studies have shown that ROS generation is essential for anthraquinone-induced apoptosis in cancer cells [51]–[52]. ROS is a potent inducer of oxidation injury that can result in base pairs oxidation as well as depurinated and aldehydic DNA [44], [46], [48], despite this injury can be counter balanced by antioxidants, whose expression are mediated by Toll pathway through ROS evaluation [53]. ROS also participate in the regulation of special cell functions, such as apoptosis [54]. ROS regulate p38 Mitogen-activated protein kinases (MAPK) phosphorylation to activate cellular apoptosis [55]–[57]. After activating MAPKKKs, such as Apoptosis signal-regulating kinase 1 (ASK1), ROS continue to phosphorylate downstream MKK3/6 to activate p38 MAPK [51]. Phosphorylated p38 MAPK then activates the mitochondrial apoptotic pathway to induce apoptosis [27], [58]. In this study, we observed that G503 enhances the ROS levels in SGC7901 cells in a time- and dose-dependent manner (Figure S2A); however, the ROS scavenger NAC do not inhibit G503-induced activation of caspase-9 and-3 as well as subsequent apoptosis (Figure S2B, C, D). Moreover, G503 increases the level of p-p38 MAPK in a time- and dose-dependent manner (Figure S3A, B). Nevertheless, NAC did not reverse p38 MAPK up-regulation induced by G503 (Figure S3C). In addition, the p38 MAPK inhibitor SB203580 do not inhibit G503-induced activation of caspase-9 and -3 nor apoptosis (Figure S3D, E, F). Although G503 promotes the generation of ROS and activates p38 MAPK, these factors are not involved in G503-induced apoptosis. G503-induced SGC7901 gastric cancer cell apoptosis is not dependent on the ROS-p38 MAPK pathway.

Caspase-9 is most commonly activated when Cyto *c*, Apaf-1 and the caspase-9 proenzyme form a complex. In addition, caspase-4 also directly activates caspase-9 via the mitochondria [29], [59]. Caspase-4 is an inflammation caspase of the Caspase-1 family that localizes to the endoplasmic reticulum(ER). Bian et al. found that the induction of ER stress by tunicamycin and thapsigargin increases the expression of caspase-4 mRNA and activation of caspase-4 [60]. In addition, Yamamuro et al. found that caspase-4 directly cleaves the caspase-9 proform at ASP-315, which is the site cleaved by the apoptosome in the mitochondrial apoptotic pathway [29]. In our study, G503 clearly activates caspase-4 (Figure 6A), and caspase-9 cleavage was not completely inhibited by the caspase-4 inhibitor Z-LEVD-FMK; the cleavage fragments of Caspase-9 remained the same, whereas the caspase-9 proform exclusively increased (Figure 6B). This result may be attributed to the fact that G503 activates caspase-9 primarily through the mitochondrial apoptotic pathway, and caspase-4 mediated activation of caspase-9 plays a small part role in G503-induced apoptosis.

**Figure 7:**
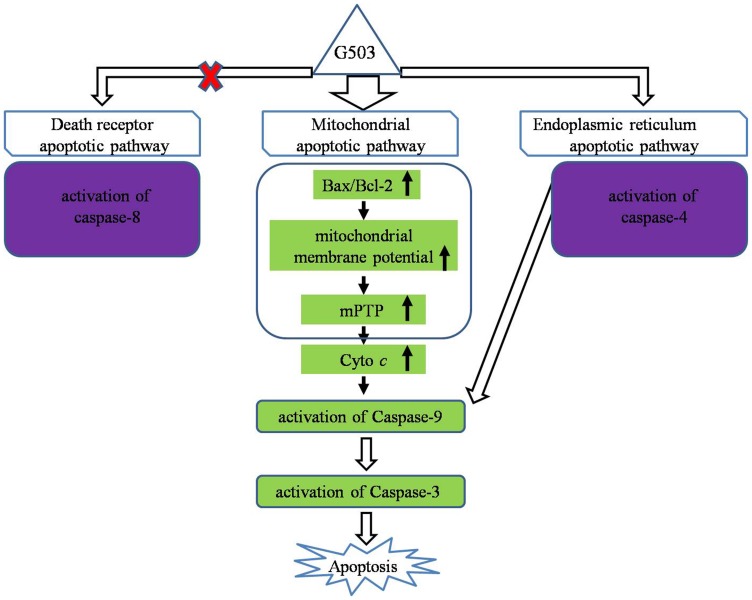
A Schematic representation of the major molecular mechanisms of G503-induced apoptosis.

In conclusion, G503, a secondary metabolite of marine microorganisms, is a potent inducer of apoptosis in SGC7901 and AGS gastric cancer cells. G503 induces apoptosis in SGC7901 gastric cancer cells primarily through the mitochondrial apoptotic pathway and partly through the activation of caspase-4 in the endoplasmic reticulum (Figure 7). G503 research suggests that it is a lead compound for the treatment of gastric cancer.

## Supporting Information

Figure S1 **Chemical structure of G503 (MW: 320).**
(TIF)

Figure S2 **G503 induces SGC7901 cells apoptosis in an ROS-independent manner.** (A) SGC7901 cells were treated with 20 µmol/L G503 for 0, 4, 12, or 24 h or various concentrations of G503 (0–40 µmol/L) for 12 h. After treatment, ROS was measured using carboxy-H2DCFDA and flow cytometry. ROS generation increased as time and concentration increased. (B–C) SGC7901 cells were pre-incubated with 5 mmol/L NAC for 2 h to prevent ROS generation and then treated with 20 µmol/L G503 for 24 h. The cells were collected, and the total protein extracts were used to detect the levels of the proform and cleaved fragments of caspase-9 and -3 by Western blotting. (D) SGC7901 cells were pre-incubated with 5 mmol/L NAC for 2 h to prevent the generation of ROS and then treated with 20 µmol/L G503 for 24 h. The apoptotic cell rate was detected by AnnexinV/PI and flow cytometry. All values are displayed as the mean ± SD of at least three independent experiments; * and ** denote p<0.05 and p<0.01, respectively.
(TIF)

Figure S3 **G503 induces SGC7901 cell apoptosis in a p38 MAPK-independent manner.** (A) SGC7901 cells were treated with 20 µmol/L G503 for various times (0.5, 6, 12, or 24 h). The cells were collected, and the total protein extracts were used to detect p38 MAPK and p-p38 MAPK levels by Western blotting. The same membrane was stripped and incubated with an antibody against β-actin for normalization. (B) SGC7901 cells were treated with G503 at various concentrations (0–40 µmol/L) for 24 h. The cells were collected, and the total protein extracts were used to detect p38 MAPK and p-p38 MAPK levels by Western blotting as described in Figure S2A. (C) SGC7901 cells were pre-treated with 5 mmol/L NAC for 2 h to prevent ROS generation and then treated with 20 µmol/L G503 for 6 h. The cells were collected, and the p38 and p-p38 levels were assessed by Western blotting. (D–E) SGC7901 cells were pre-incubated with 10 µmol/L of the p38 MAPK inhibitor (SB203580) for 1 h and then treated with 20 µmol/L G503 for 24 h. The cells were collected, and the total protein extracts were used to detect caspase-9 and -3 levels by Western blotting. (F) The cells were pre-incubated with the p38 MAPK inhibitor (SB203580) for 1 h and then treated with 20 µmol/L G503 for 24 h. The apoptotic cell rate was determined by Annexin V/PI and flow cytometry. All values are displayed as the mean ± SD of at least three independent experiments (*P<0.05, **P<0.01 vs. control).
(TIF)

File S1 **G503 induces apoptosis in a ROS-MAPK-independent manner.**
(DOC)
