# Supplementary figures and images for: Anthraquinone G503 Induces Apoptosis in Gastric Cancer Cells through the Mitochondrial Pathway
Source: PLoS One. 2014 Sep 30;9(9):e108286. doi: 10.1371/journal.pone.0108286 (PMC4182468; doi:10.1371/journal.pone.0108286)

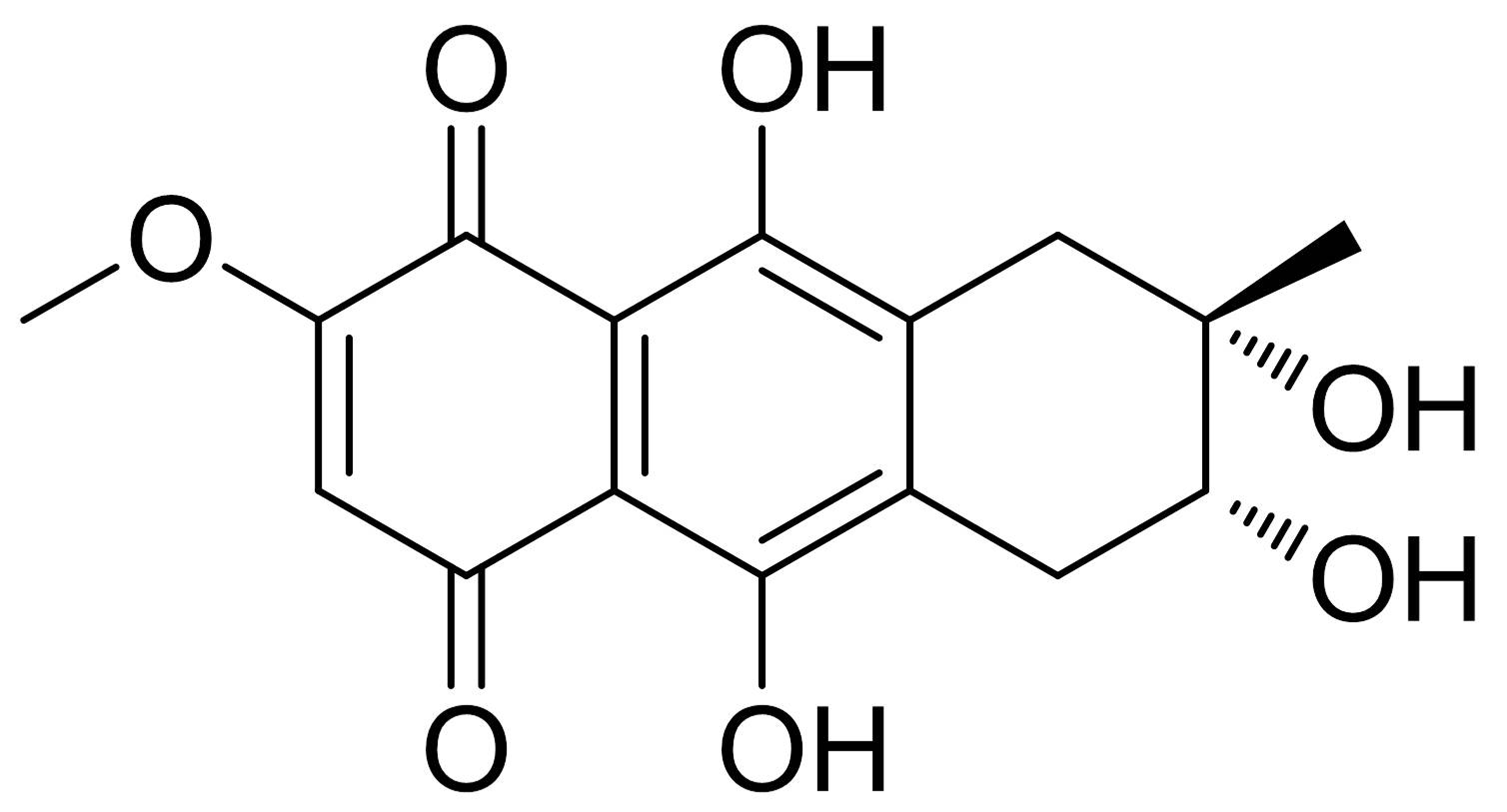

Supplement: Figure S1 — Chemical structure of G503 (MW: 320). (TIF) [file pone.0108286.s001.tif]

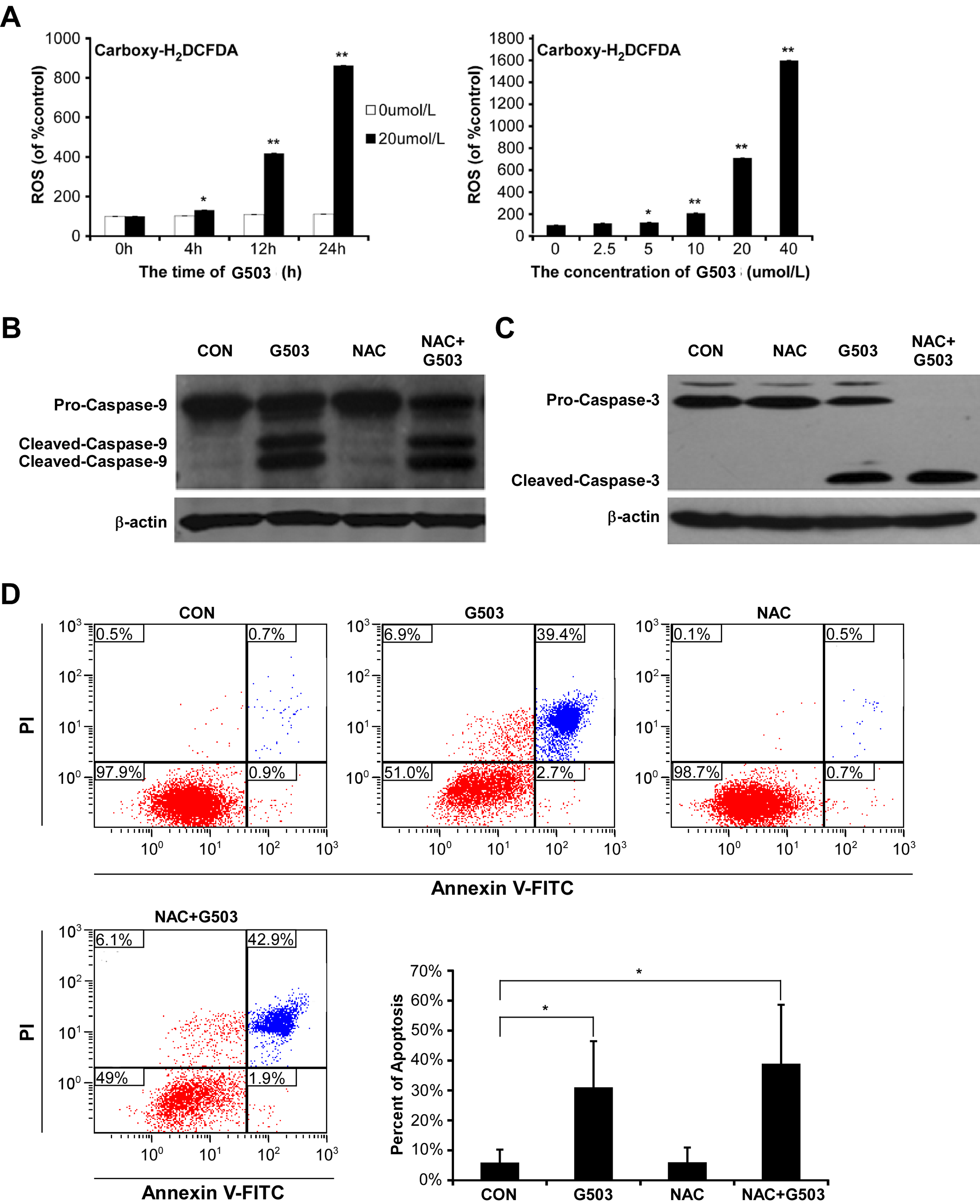

Supplement: Figure S2 — G503 induces SGC7901 cells apoptosis in an ROS-independent manner. (A) SGC7901 cells were treated with 20 µmol/L G503 for 0, 4, 12, or 24 h or various concentrations of G503 (0–40 µmol/L) for 12 h. After treatment, ROS was measured using carboxy-H2DCFDA and flow cytometry. ROS generation increased as time and concentration increased. (B–C) SGC7901 cells were pre-incubated with 5 mmol/L NAC for 2 h to prevent ROS generation and then treated with 20 µmol/L G503 for 24 h. The cells were collected, and the total protein extracts were used to detect the levels of the proform and cleaved fragments of caspase-9 and -3 by Western blotting. (D) SGC7901 cells were pre-incubated with 5 mmol/L NAC for 2 h to prevent the generation of ROS and then treated with 20 µmol/L G503 for 24 h. The apoptotic cell rate was detected by AnnexinV/PI and flow cytometry. All values are displayed as the mean ± SD of at least three independent experiments; * and ** denote p<0.05 and p<0.01, respectively. (TIF) [file pone.0108286.s002.tif]

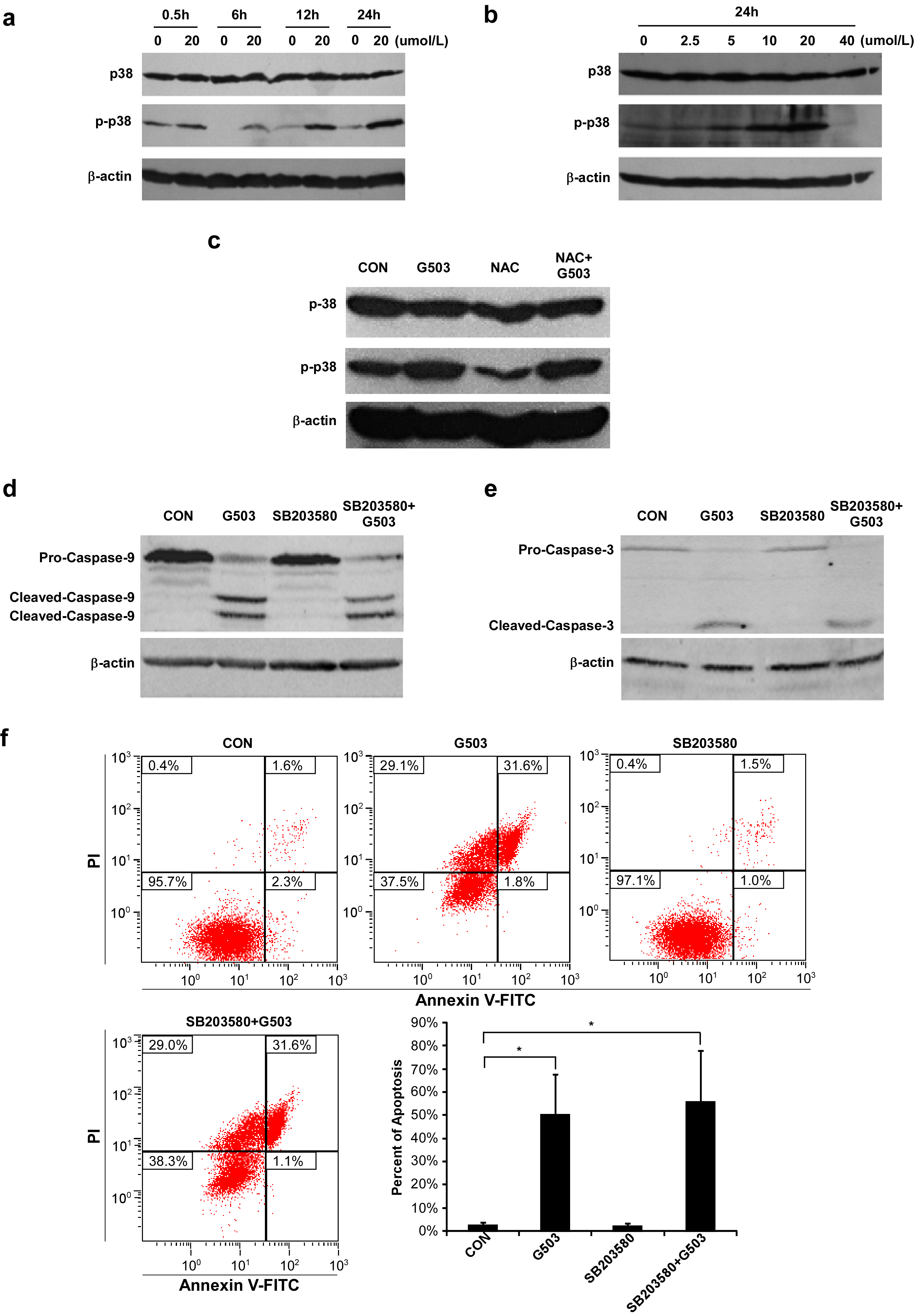

Supplement: Figure S3 — G503 induces SGC7901 cell apoptosis in a p38 MAPK-independent manner. (A) SGC7901 cells were treated with 20 µmol/L G503 for various times (0.5, 6, 12, or 24 h). The cells were collected, and the total protein extracts were used to detect p38 MAPK and p-p38 MAPK levels by Western blotting. The same membrane was stripped and incubated with an antibody against β-actin for normalization. (B) SGC7901 cells were treated with G503 at various concentrations (0–40 µmol/L) for 24 h. The cells were collected, and the total protein extracts were used to detect p38 MAPK and p-p38 MAPK levels by Western blotting as described in Figure S2A. (C) SGC7901 cells were pre-treated with 5 mmol/L NAC for 2 h to prevent ROS generation and then treated with 20 µmol/L G503 for 6 h. The cells were collected, and the p38 and p-p38 levels were assessed by Western blotting. (D–E) SGC7901 cells were pre-incubated with 10 µmol/L of the p38 MAPK inhibitor (SB203580) for 1 h and then treated with 20 µmol/L G503 for 24 h. The cells were collected, and the total protein extracts were used to detect caspase-9 and -3 levels by Western blotting. (F) The cells were pre-incubated with the p38 MAPK inhibitor (SB203580) for 1 h and then treated with 20 µmol/L G503 for 24 h. The apoptotic cell rate was determined by Annexin V/PI and flow cytometry. All values are displayed as the mean ± SD of at least three independent experiments (*P<0.05, **P<0.01 vs. control). (TIF) [file pone.0108286.s003.tif]
