# Supplementary material for: Anthraquinone G503 Induces Apoptosis in Gastric Cancer Cells through the Mitochondrial Pathway
Source: PLoS One. 2014 Sep 30;9(9):e108286. doi: 10.1371/journal.pone.0108286 (PMC4182468; doi:10.1371/journal.pone.0108286)
Supplement: File S1 — G503 induces apoptosis in a ROS-MAPK-independent manner. (DOC) [file pone.0108286.s004.doc]

**Supporting Information**

**Materials and Methods**

**Reactive oxygen species (ROS) measurement**

SGC7901 cells were seeded into 6-well plates. When to the cells achieved 60-70% confluency, the cells were treated with G503 concentrations ranging from 0 to 40 µmol/L for 23 h. Some cells were pretreated with 5 mmol/L N-acetyl-cysteine (NAC) for 2 h. The cells were incubated with 10 µmol/L of the ROS fluorescent probe carboxy-H2DCFDA at 37°C for 1 h in the dark. After treatment, the cells were collected by trypsin digestion and centrifuged at 600 g for 5 min. The cells were resuspended in PBS and measured by flow cytometry.

**Results and Discussion**

**G503-induced apoptosis in SGC7901 cells is ROS-independent**

Anthraquinone generates ROS, which participate in the regulation of special cell functions, such as apoptosis. Therefore, we used the fluorescent probe carboxy-H_2_DCFDA to determine ROS levels in G503-treated cells. After SGC7901 cells were incubated with G503 at various concentrations for indicated times, we observed that G503 increased ROS levels in a time- and dose-dependent manner (Figure S2A).

To further investigate the role of G503-induced ROS in apoptosis, the ROS scavenger NAC was employed to inhibit ROS formation. SGC7901 cells were treated with 20 µmol/L G503 for 24 h after pretreatment with 5 mmol/L NAC for 2 h. The cells were collected to detect caspase-9 and -3 by Western blotting and to quantify the number of apoptotic cells through Annexin V-FITC/PI staining and flow cytometry. Compared with the drug only group, the caspase-9 and -3 proform levels remained low and the activated cleavage fragments of the two caspases remained enhanced after treatment with NAC (Figure S2B, C). Consistent with the caspase-9 and -3 results, NAC pretreatment did not decrease the apoptotic cell rate (Figure S2D). These data suggest that NAC does not prevent the activation of caspase-9 and -3 as well as apoptosis in SGC7901 cells. Taken together, the data indicate that G503-induced apoptosis is not dependent on ROS.

**G503-induced apoptosis in SGC7901 cells is ROS-p38 MAPK-independent**

p38 MAPK can be phosphorylated by ROS, and p-p38 MAPK induces apoptosis through the activation of the mitochondrial apoptotic pathway. To examine whether p38 MAPK is involved in G503-induced apoptosis in SGC7901 cells, the cells were treated with G503 at various concentrations for indicated times, and p38 MAPK and p-p38 MAPK protein levels were detected by Western blotting. Figure S3A,B indicates that p38 levels were not significantly altered with various doses of G503; however, the levels did decrease with increasing exposure to G503. In contrast, p-p38 levels increased in a time- and dose-dependent manner. However, when SGC7901 cells were co-treated with G503 and the ROS scavenger N-acetyl-cysteine (NAC), no changes were observed in the p-38 and p-p38 levels compared with the G503 group (Figure S3C). Therefore, ROS did not activate p-p38. When p38 MAPK was inhibited by SB203580 in SGC7901 cells, the Western blotting results indicate that SB203580 does not inhibit G503-mediated decreases in the caspase-9 and -3 proformsnor increases in the cleavage fragments of the two caspases (Figure S3D, E). Consistent with the caspase-9 and -3 results, the flow cytometry results indicate that SB203580 does not decrease the apoptotic cell rate induced by G503 in SGC7901 cells pre-incubated with 10 µmol/L SB203580 for 1 h and co-treated with 20 µmol/L G503 for 24 h (Figure S3F). These data suggest that G503-induced apoptosis in SGC7901 gastric cancer cells is not dependent on the activation of ROS-p38 MAPK.

**References**

1. Bolton JL, Trush MA, Penning TM, Dryhurst G, Monks TJ (2000) Role of quinones in toxicology. Chem Res Toxicol 13: 135-160.

2. Wang X, Thomas B, Sachdeva R, Arterburn L, Frye L, et al. (2006) Mechanism of arylating quinone toxicity involving Michael adduct formation and induction of endoplasmic reticulum stress. Proc Natl Acad Sci U S A 103: 3604-3609.

3. Danial NN, Korsmeyer SJ (2004) Cell death: critical control points. Cell 116: 205-219.

4. Junttila MR, Li SP, Westermarck J (2008) Phosphatase-mediated crosstalk between MAPK signaling pathways in the regulation of cell survival. Faseb j 22: 954-965.

5. Selimovic D, Hassan M, Haikel Y, Hengge UR (2008) Taxol-induced mitochondrial stress in melanoma cells is mediated by activation of c-Jun N-terminal kinase (JNK) and p38 pathways via uncoupling protein 2. Cell Signal 20: 311-322.

6. Zielinska-Park J, Nakamura J, Swenberg JA, Aitken MD (2004) Aldehydic DNA lesions in calf thymus DNA and HeLa S3 cells produced by bacterial quinone metabolites of fluoranthene and pyrene. Carcinogenesis 25: 1727-1733.
